# Patterns of gastrointestinal pathogen co-detection in pediatric stool samples identified by rapid multiplex PCR

**DOI:** 10.1017/S0950268826101083

**Published:** 2026-02-04

**Authors:** Keyao Xiong, Juliana M. Ruzante, Ross M. Maltz, James Andrew Barkley, Nadira Yasmin, Devin LaPolt, Tyler Cobb, Barbara Kowalcyk

**Affiliations:** 1 Department of Food Science and Technology, The Ohio State University, Center for Foodborne Illness Research and Prevention, USA; 2 RTI International, USA; 3 Division of Pediatric Gastroenterology, Hepatology and Nutrition, Nationwide Children’s Hospital, USA; 4 Department of Pediatrics, The Ohio State University Wexner Medical Center, USA; 5 Food Science and Technology, The Ohio State University, USA; 6 The George Washington University Milken Institute School of Public Health, USA

**Keywords:** acute gastrointestinal infections (AGI), co-detections, culture-independent diagnostic tests (CIDTs), epidemiology, gastrointestinal pathogens, pediatrics

## Abstract

Acute gastrointestinal infections (AGIs) can lead to significant morbidity and mortality. In diagnosing AGI, culture-independent diagnostic tests offer advantages over traditional methods and increase the chance of detecting multiple pathogens (co-detection). A retrospective analysis of data from a tertiary pediatric hospital was conducted to characterize occurrence of AGI co-detections and compare outcomes with patients who had only one AGI pathogen detected. Medical records were obtained for patients with stool samples tested using BioFire FilmArray GI Panel between 1 January 2016 and 31 December 2020. Data were described using descriptive statistics, correlation analysis, and logistic regression to identify risk factors and estimate co-detection rates. During the study period, 12,753 patients had a total of 17,159 stool samples tested. Of these, 8,212(47.9%) tested positive, with 6,040(73.6%) being single detections and 2,172(26.4%) being co-detections. Patients with single detection experienced higher hospitalization rates than patients with co-detection. Patients 1–4 years old exhibited the highest co-detection rate relative to other age groups, while Hispanic/Latino individuals were 1.75 times more likely to have co-detection than other races. This study emphasizes the significance of understanding pathogen interactions concerning clinical characteristics and epidemiology of AGI, and the necessity for effective diagnostic strategies and optimal healthcare resource allocation.

## Introduction

Acute gastrointestinal infections (AGIs) are a significant public health concern, with children bearing most of the disease burden [1]. Prevention of AGI through good hygiene and access to safe food and water is the most efficient way to protect public health; however, when illnesses present with features that warrant diagnostic evaluation, targeted and timely testing can be important for effective patient care and for detecting potential outbreaks. In clinical settings, selective testing may guide antibiotic use, avoid unnecessary hospitalizations, and inform isolation decisions, thereby reducing healthcare expenditures [2–4]. Accurate detection is also critical to surveillance of AGI pathogens, which is used to inform the development of targeted interventions to control pathogens, monitor trends in incidence, and estimate the burden of disease [5].

A range of infectious agents, including bacteria, parasites, and viruses, can cause AGI. Physicians can order stool samples for patients presenting with AGI symptoms and request bacterial culture, ova and parasite examinations, and targeted viral assays to identify the causative organism and inform treatment. However, detecting the cause of illness can be challenging and expensive given the range of possible pathogens, lack of pathogen-specific symptoms, and difficulties in culturing some AGI pathogens [3, [6], 7]. Often, these tests are highly sensitive, faster, and generally more cost-effective than traditional culture-based methods [3, 4]. As a result, many clinical laboratories have shifted to the use of molecular-based culture-independent diagnostic tests (CIDTs) that can detect multiple pathogens in a single stool sample test.

With the ability to test multiple pathogens simultaneously and the improved detection sensitivity, CIDTs are also increasing the detection of multiple pathogens in one single stool sample (i.e., co-detection). The rates of co-detection of AGI pathogens in the general population have been reported in several studies, varying from around 8% to 30% of positive results [6, 8]. However, these studies were limited to shorter time frames and focused on the general population. The rates of co-detection in the pediatric population have been reported as ranging from 15% to 65% [9, 10]. The clinical significance of multiple pathogen detections remains unclear. Co-detections may reflect higher pathogen burden, distinct transmission patterns, or underlying host susceptibility, any of which could influence symptom severity, dehydration risk, or healthcare utilization. However, few studies have evaluated whether children with co-detection events experience different clinical outcomes, such as hospitalization, compared with those with single detections [11–13]. Therefore, the objective of this research was to characterize the occurrence of co-detections of AGI pathogens in a pediatric population and compare patient characteristics and outcomes between those with single detections and those with co-detections.

## Materials and methods

Electronic medical records were collected from Nationwide Children’s Hospital (NCH) in Columbus, Ohio, for patients under the age of 25 with at least one stool sample tested between 1 January 2016 and 31 December 2020. This analysis represents a secondary evaluation of the dataset described in Yasmin et al. [14]. For the current analysis, we restricted the cohort to patients with a single FilmArray test during the encounter and complete hospitalization data. It was assumed that patients with multiple tests during the study period might represent those with prolonged illness, recurrent infections, or patients with more severe or medically complex conditions. As a result, the number of patients included here is a subset of the broader cohort reported previously. The present manuscript focuses specifically on the clinical significance of co-infections, which were not examined in the earlier analysis.

During the study period, NCH tested stool samples using the BioFire® FilmArray® Gastrointestinal (GI) Panel (Salt Lake, Utah), a commercial CIDT that uses real-time polymerase chain reaction to detect 22 AGI pathogenic targets, including 13 bacteria, 5 viruses, and 4 parasites, in human stool samples [6, 15]. Testing was not performed universally but was ordered when the provider deemed diagnostic evaluation appropriate. FilmArray testing was performed at or near the time of clinical presentation, and hospitalization status reflects whether the patient was admitted during that same encounter. Thus, testing and hospitalization occurred within the same clinical episode. De-identified demographic information (i.e., age, race and ethnicity, sex, zip code, insurance type), laboratory results (i.e., order date, test result), as well as healthcare encounter data (i.e., encounter date, diagnostic code, outcome) were extracted for each patient as described elsewhere [14]. NCH is a free-standing children’s hospital that routinely provides care to infants, children, and young adults. We restricted the study population to patients ≤25 years of age. Individuals older than 25 at this institution typically have complex chronic conditions and differ clinically from the general pediatric and young adult population. Urbanicity was defined using the patient’s zip code and assigned using Rural–Urban Commuting Area (RUCA) codes from the U.S. Department of Agriculture Economic Research Service (USDA ERS) mapping file [16]. Zip codes were matched to RUCA codes and grouped into the urbanicity categories presented in Table 1. Patients were categorized as having public insurance if billing records indicated Medicare, Medicaid, or the Children’s Health Insurance Program (CHIP). Hospitalization was defined as inpatient admission or observation status occurring during the same encounter in which the FilmArray test was ordered. Emergency department visits without admission were not considered hospitalizations. Because observation encounters are recorded with short lengths of stay, some admissions had durations of <1 day.

**Table 1.** Patient characteristics overall and by detection type

	Patients*	FilmArray Tests – N (%)			
	Total	Total	Single Detection N (%)	Co-detection	*P*-value**
Total	12,753 (100%)	17,159 (100%)	6,040 (35.2%)	2,172 (12.7%)	
Hospitalizations	6,673 (52.3%)	9,732 (56.7%)	3,403 (56.34%)	1,132 (52.1%)	<0.01
Deaths	48 (0.4%)	48***	18 (0.3%)	2 (0.1%)	0.09
Age group					
<1	2,194 (17.2%)	2,901 (16.9%)	1,016 (16.8%)	410 (18.9%)	<0.01
1–4	4,193 (32.9%)	5,533 (32.3%)	2,299 (38.1%)	1,045 (48.1%)	
5–12	3,120 (24.5%)	4,118 (24.0%)	1,443 (23.9%)	441 (20.3%)	
13–18	2,613 (20.5%)	3,680 (21.4%)	1,033 (17.1%)	220 (10.1%)	
18–25	633 (5.0%)	927(5.4%)	249 (4.1%)	56 (2.6%)	
Sex					
Female	5,948 (46.7%)	8,002 (46.6%)	2,732 (45.2%)	981 (45.2%)	0.96
Male	6,805 (53.3%)	9,157 (53.4%)	3,308 (54.8%)	1,191 (54.8%)	
Race & ethnicity					
White Non-Hispanic	8,166 (64.0%)	11,238 (65.5%)	3,946 (65.3%)	1,216 (56.0%)	<0.01
Black Non-Hispanic	2,247 (17.6%)	2,846 (16.6%)	1,019 (16.9%)	463 (21.3%)	
Asian	474 (3.7%)	606 (3.5%)	209 (3.5%)	108 (5.0%)	
Hispanic or Latino	646 (5.1%)	773 (4.5%)	288 (4.8%)	156 (7.2%)	
Other	1,220 (9.6%)	1,696 (9.9%)	578 (9.6%)	229 (10.5%)	
Insurance type					
Public (Medicaid/CHIP)	6,376 (50%)	8,612 (50.2%)	3,074 (50.9%)	1,299(59.8%)	<0.01
Private	4,927 (38.6%)	6,233(36.3%)	2,218 (36.7%)	653 (30.1%)	
Self-pay	259 (2%)	300 (1.8%)	105 (1.7%)	65 (3.0%)	
Combination Public/Private	1,160 (9.1%)	1,950 (11.4%)	621 (10.3%)	153 (7.0%)	
Other or Unknown	31 (0.2%)	64 (0.4%)	22 (0.4%)	2 (0.1%)	
Urbanicity					
Metropolitan (Urban Area)	9,746 (76.4%)	12,820 (74.7%)	4,529 (75.0%)	1,682 (77.4%)	0.15
Micropolitan (Large Urban Cluster)	2,398 (18.8%)	3,464 (20.1%)	1,211 (20.1%)	385 (17.7%)	
Small Town (Small Urban Cluster)	422(3.3%)	615 (3.6%)	208 (3.4%)	78 (3.6%)	
Rural Area	147 (1.2%)	183 (1.1%)	68 (1.1%)	21 (1%)	
Missing Zip Code	40 (0.3%)	77 (0.5%)	24 (0.4%)	6 (0.3%)	

* Pearson’s Chi-square tests were used to test for different demographic groups.

** The p-values were calculated for each category for the overall FilmArray® GI Panel tests between the single detection and co-detection populations.

*** The overall number of patient deaths is 48, despite the number of tests conducted on each patient.

Patient comorbidities were summarized using the Charlson Comorbidity Index (CCI), which classifies conditions by assigning predetermined numeric values to patients (Charlson et al., 1987). The CCI was used as a standardized measure of baseline health status because it was the only validated comorbidity metric that could be reliably derived from the structured data available for this cohort. In brief, International Classification of Diseases (ICD) codes were extracted for each patient, and CCI weights were used to score each diagnosis. For example, more prevalent conditions, such as myocardial infarction or diabetes, were assigned 1 point, while moderate/severe illnesses, such as liver disease, received 3 points, and metastatic solid tumors or AIDS, which are considered very severe, received 6 points, as determined by the CCI. Scores were summed to produce a total CCI score for each patient, which was then categorized as mild (1–2 points), moderate (3–4 points), or severe (≥5 points) comorbidities according to the CCI standard [17].

Patient characteristics (age, sex, race and ethnicity, urbanicity, insurance type) and outcomes (hospitalization, mortality) were summarized using descriptive statistics. Rates of single and co-detection were estimated, along with 95% confidence intervals, by dividing the number of single or co-detections (i.e., two or more AGI pathogens detected in the same sample) by the total number of FilmArray tests performed. Co-detection rates were estimated by pathogen by dividing the total number of co-detections for each pathogen by the total number of co-detections. A correlation analysis was conducted to identify AGI pathogens commonly detected together. *Escherichia coli* O157 and Shiga toxin-producing *E. coli* (STEC) were combined for analysis. *Vibrio* and *Vibrio cholerae* were combined due to low detection numbers.

Logistic regression was used to identify risk factors associated with co-detection, hospitalization, and mortalitySince deaths were rare in this cohort, analyses of mortality were not powered to detect differences between infection groups and are interpreted descriptively. Risk factors considered included age, sex, race and ethnicity, urbanicity, insurance type, co-detection (for hospitalization and mortality models), and comorbidity status. Since individual pathogen combinations were infrequent, co-infections were categorized as a single binary variable (≥2 pathogens detected). This approach was used to maintain adequate cell counts, avoid model instability due to sparse categories, and evaluate the broader clinical relevance of multiple simultaneous detections. The outcomes of interest were co-detections, hospitalization, and mortality. For each model, backward selection was used to evaluate the relationship between risk factors and the outcome of interest. Crude and adjusted odds ratios and 95% confidence intervals were calculated for potential risk factors, and a p-value ≤0.05 was considered statistically significant. Pairwise comparisons with Bonferroni adjustments were estimated for the outcomes of interest. Goodness-of-fit was assessed using the Hosmer-Lemeshow goodness-of-fit test. Multicollinearity was assessed by examining pairwise correlations among continuous predictors, evaluating low-frequency levels of categorical variables, and reviewing model diagnostics (including condition indices). Variance Inflation Factors (VIFs) were calculated for continuous variables where applicable. Hospitalization and mortality rates were estimated by dividing the number of hospitalized patients or the number who had died by the total number of patients and stratified by demographics. All statistical analyses were performed using SAS 9.4 for Windows (SAS Institute, Cary, NC). Appropriate ethical approvals were obtained from NCH [STUDY00001605], The Ohio State University [2021 N0015], and George Washington University [NCR235216].

## Results

**Figure 1.**
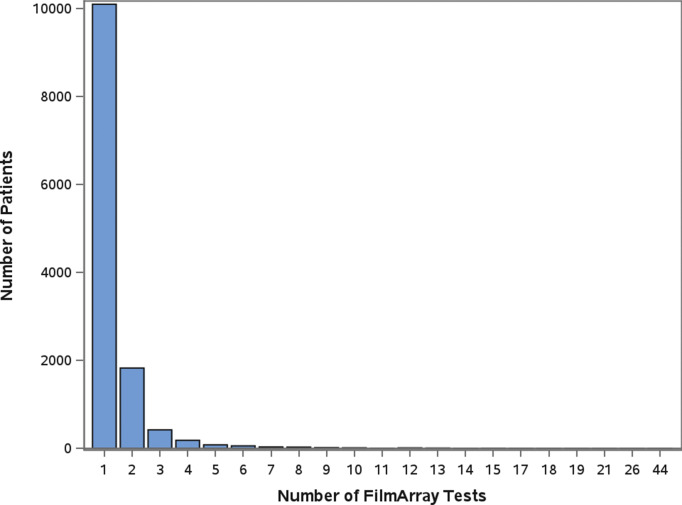
Number of FilmArray Tests by patient (N = 12,753).

**Figure 2.**
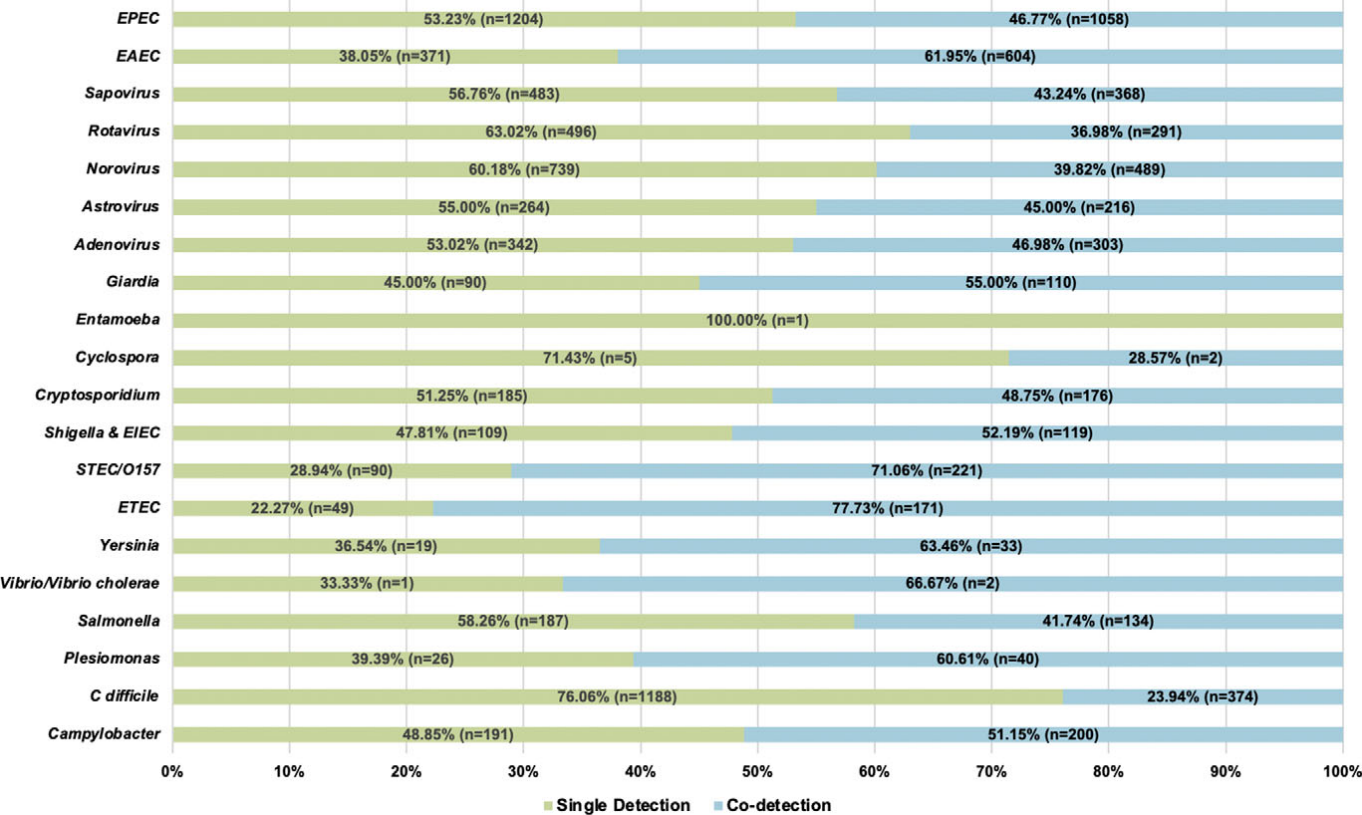
Number of detections and proportion of single and co-detections by pathogen (N = 17,159).

**Figure 3.**
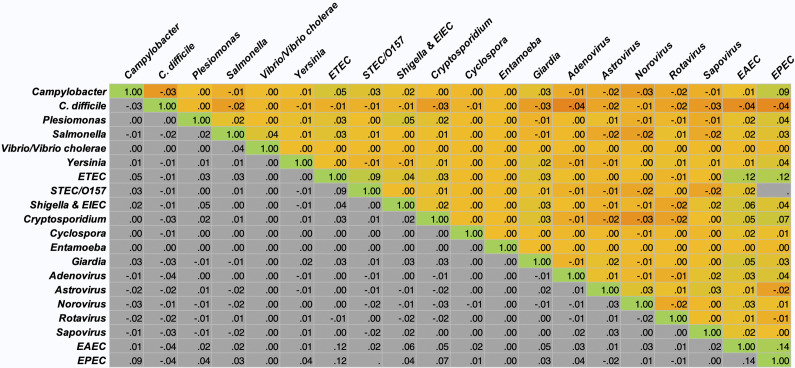
Pathogen Co-detection Correlation Heat Matrix.

A total of 12,753 patients had a stool sample tested using the GI FilmArray from January 1, 2016, to December 31, 2020, at NCH (Table 1); 30 patients with over 10 stool sample tests during the study period were considered outliers and excluded from the analysis [14]. The mean age was 7.1 years, with just over half (50.1%) below the age of 5. Patients were predominantly male (53.3%), white non-Hispanic (64%), lived in a metropolitan area (76.4%), and had public insurance (50%). Most patients (99.9%) did not have a comorbidity and, therefore, did not have a CCI score. Out of the patients with comorbidities recorded (N = 315), 295 were classified as mild, and 20 were considered severe. Patients had 17,159 stool samples tested during the study period, with the majority (79%) having one test (Figure 1). A total of 8,212 (47.9%) tests detected at least one AGI pathogen, with a single pathogen being detected in 35.2% and multiple pathogens being detected in 12.7% (Table 1). Pathogens most commonly detected were Enteropathogenic *E. coli* (EPEC), *Clostridioides difficile*, and norovirus. Co-detection rates of 60% or higher were observed for *E. coli* O157/STEC, Enterotoxigenic *E. coli* (ETEC), Vibrio, Yersinia, Enteroaggregative *E. coli* (EAEC), and *Plesiomonas shigelloides* (Figure 2) [14]. An alternative figure presenting co-detection rates in unique patients can be found in Supplementary Figure 1. *C. difficile* and *Cyclospora cayetanensis* exhibited lower than 30% co-detection rates. Numerous weak but statistically significant correlations were identified for co-detections (p-value <0.0001) (Figure 3). Positive correlations were observed between EPEC and EAEC (0.14), EPEC and ETEC (0.12), *E. coli* O157/STEC and ETEC (0.09), and EPEC and Campylobacter (0.08). In contrast, *C. difficile* was negatively associated with several other AGI pathogens [e.g., Adenovirus (0.04), EPEC (0.04), and EAEC (0.04)]. Among co-infected children, pathogen combinations were highly heterogeneous and often rare; the most frequently observed combinations and their associated hospitalization rates are shown in Supplementary Table 1.

**Figure 4.**
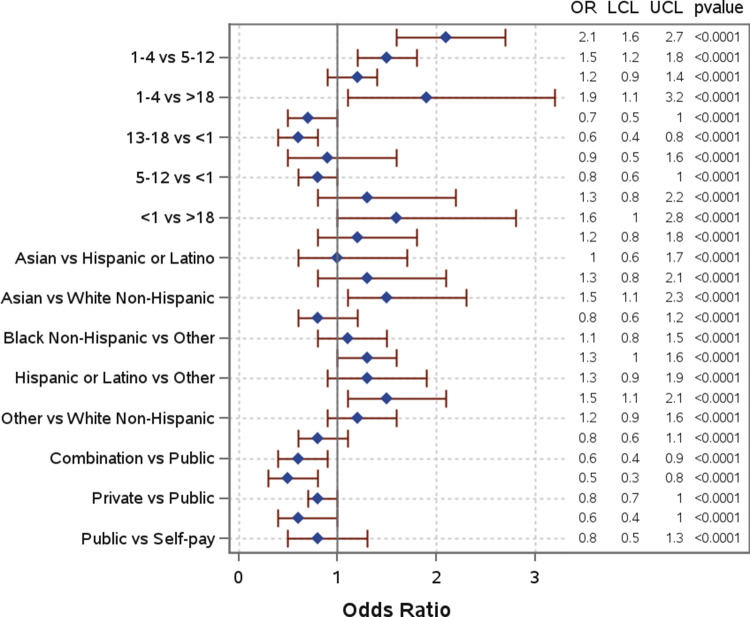
Odds of co-detection by demographic in single and co-detection groups.

Several sociodemographic characteristics were associated with co-detection of pathogens (Table 1, Figure 4). Children aged 1–4 years had significantly higher odds of co-detections among all age groups and were significantly more likely to have co-detections compared to children aged 13–18 years (AOR: 2.1; 95% CI: 1.6–2.7) and those over 18 years (AOR: 1.9; 95% CI: 1.1–3.2). When compared to White non-Hispanic individuals, Asian, Black non-Hispanic, and Hispanic or Latino individuals were also significantly more likely to have co-detections, with Asian (AOR: 1.5; 95% CI: 1.1–2.3) and Hispanic or Latino individuals (AOR: 1.5; 95% CI: 1.1–2.1) having the highest odds. Individuals with a combination of public and private insurance had significantly lower odds of co-detections compared to individuals with public insurance (AOR: 0.6; 95% CI: 0.4–0.9) and those who self-pay (AOR: 0.5; 95% CI: 0.3–0.8). Additional model output can be found in Supplementary Table 2.

**Figure 5.**
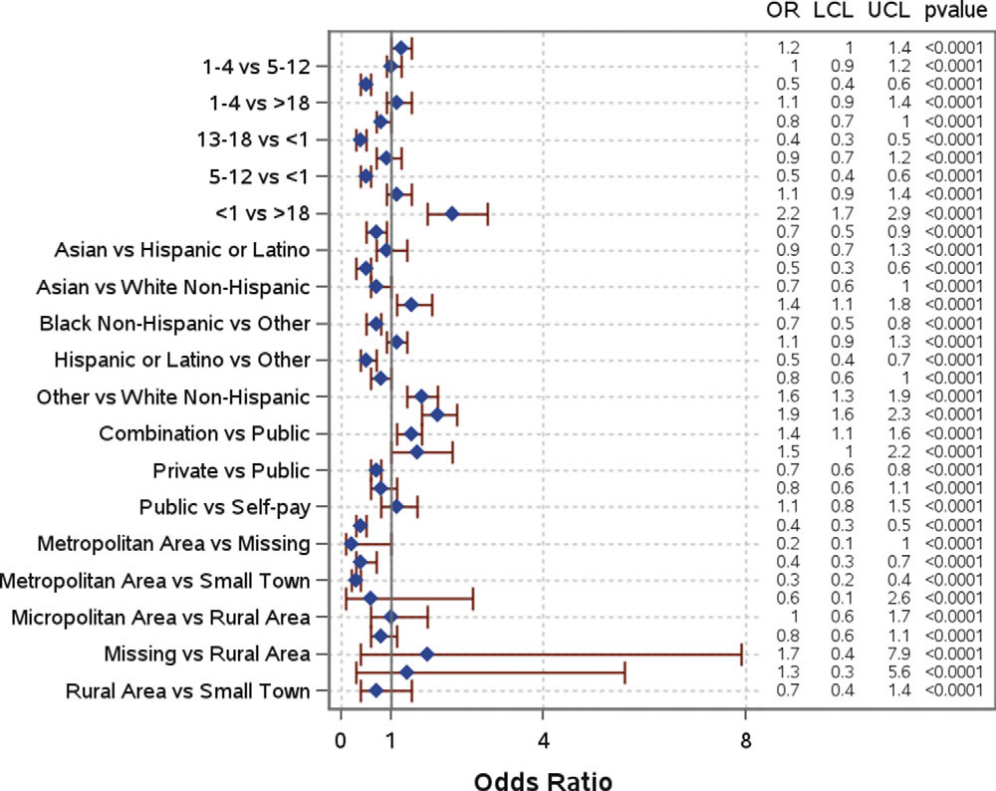
Odds of hospitalization by demographic in total patient population.

Slightly more than half of the patients (52.3%) were hospitalized (Table 1). The mean and median number of days in the hospital were, respectively, 4.8 and 1 days for patients with a single pathogen detection, and 2.3 and 0.5 days for patients with a co-detection. Several risk factors were significantly associated with hospitalization (Figure 5). Children under 1 year old had significantly higher odds of hospitalization compared to all other age groups and were more than twice as likely compared to those over 18 years (AOR: 2.2; 95% CI: 1.7–2.9) (Figure 5). Similarly, children aged 1–4 years had moderately higher odds of hospitalization compared to those aged 13–18 years (AOR: 1.2; 95% CI: 1.0–1.4). When compared to White non-Hispanic individuals, individuals who identified as “Other” (AOR: 1.6; 95% CI: 1.3–1.9) had significantly higher odds of hospitalization. Higher odds of hospitalization were also found for Black non-Hispanic individuals compared to Hispanic and Latino individuals (AOR: 1.4; 95% CI: 1.1–1.8). Individuals with a combination of public and private insurance had significantly higher odds of hospitalization compared to those with private insurance (AOR: 1.9; 95% CI: 1.6–2.3), public insurance (AOR: 1.4; 95% CI: 1.1–1.6), and those who self-pay (AOR: 1.5; 95% CI: 1.0–2.2). Those living in metropolitan areas had significantly lower odds of hospitalization compared to individuals living in micropolitan areas (AOR: 0.4; 95% CI: 0.3–0.5), rural areas (AOR: 0.4; 95% CI: 0.3–0.7), and small towns (AOR: 0.3; 95% CI: 0.2–0.4). Co-detection was not a statistically significant risk factor for hospitalization. Additional information can be found in Table 1 and Figure 5.

**Figure 6.**
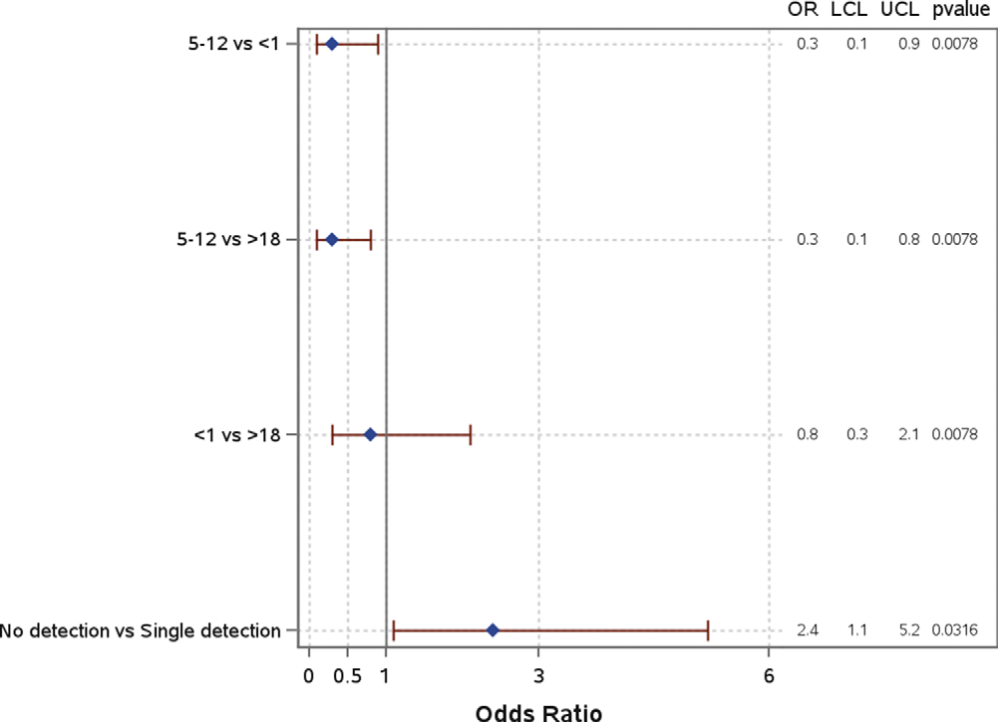
Odds of mortality by demographic in total patient population.

Age group was significantly associated with mortality (Figure 6). When compared to both children under 1 year (OR: 0.3; 95% CI: 0.1–0.9) and those over 18 years (OR: 0.3; 95% CI: 0.1–0.8), children aged 5–12 years had significantly lower odds of mortality. There was no significant difference in the odds of mortality for children under 1 compared to those over 18 years (OR: 0.8; 95% CI: 0.3–2.1). Other characteristics, including race and insurance group, were analyzed but showed no significant associations with mortality. Co-detection was not significantly associated with mortality (Supplemental Material 1).

Patients with mild comorbidities had higher odds of both single and co-detection compared to no-detection, but the p-value was only significant (p < 0.05) for those with single detection (Table 2). Patients with severe comorbidities had higher odds of single and co-detection compared to non-detection, but those were not significant (p > 0.1). There was insufficient data to assess the association with hospitalization and mortality.

**Table 2.** Logistic regression analysis for Charlson comorbidity index levels

	Co-detection								
CCI level^a^	No detection^b^			Single detection^c^			Co-detection^d^		
	OR^e^	95%CI^f^	p-value	OR	95%CI	p-value	OR	95%CI	p-value
Mild (1–2 points)	Ref^g^	-	-	0.77	(0.62, 0.97)	0.02	0.82	(0.57, 1.77)	0.27
Severe (≥5 points)	Ref	-	-	1.79	(0.80, 4.00)	0.15	1.58	(0.45, 5.53)	0.48

a Charlson Comorbidity Index.

b Individual logistic regression model.

c Individual logistic regression model.

d Individual logistic regression model.

e Odds Ratio.

f 95% Wald Confidence Interval.

g Reference group.

## Discussion

To the best of our knowledge, this is one of the first studies to describe the prevalence and epidemiological characteristics of co-detections in pediatric patients not limited to diarrheal disease, from a large tertiary pediatric hospital in the US. This study focused on assessing co-detections between a number of pathogens, while previous studies in children have been limited to small sample sizes and a few pathogens of interest [6, 8].

The observed proportions of single and co-detections aligned with findings from previous investigations of the GI Panel in clinical practice within the United States [2, [5]–[8], 18]. While EPEC, *C. difficile*, and norovirus were the most commonly detected pathogens, these pathogens were unlikely to be co-detected with another pathogen. In contrast, *E. coli* O157/STEC, ETEC*, Yersinia*, EAEC, and *Plesiomonas shigelloides* were more likely to be co-detected, which was consistent with a recent study evaluating pathogen detections that also reported co-detections between ETEC, STEC, and EAEC [19] and a European study reporting multiple *E. coli* infections in unique patient stool samples [20].

Higher odds of co-detection were seen in younger patients, non-white patients, and patients with public and self-pay insurance, which is consistent with previous research. A recent study of children with gastrointestinal infections reported high rates of co-detection; however, this study was primarily focused on viral agents [21]. A cross-sectional study conducted from 2000–2015 reported that Black and Asian/Pacific Islander populations had the highest risk of *Helicobacter pylori* infection and upper GI symptoms compared to other racial/ethnic groups [22]. However, this study was limited to adults 21–65 years old and focused on one pathogen, so comparisons between race/ethnicity and co-detection cannot be drawn. Additionally, a retrospective study conducted from 2011–2017 reported that patients with Medicaid had a higher burden of *C. difficile* [23]. However, this study was conducted in adult and elderly populations, while our study focused on children.

Co-detection did not increase odds for hospitalization or mortality; patients with more than one pathogen detection had lower rates and length of stay for hospitalization. This finding differs from a previous study, which reported that GI co-infection was associated with chronic kidney disease [19]. However, this study was primarily focused on adult patients, which differs from our study population of children. Another study found that children with co-infection from enteric pathogens had more severe clinical symptoms and dehydration [10]. Additionally, co-detection can potentially result in different host immune responses, which may limit disease severity, as has been documented in parasite, virus, and bacterial studies [24–27].

These findings also have implications for diagnostic stewardship. Multiplex gastrointestinal panels are increasingly used across clinical settings, yet their optimal role remains uncertain [28, 29]. The observed variability in pathogen profiles and the predominance of mild, self-limiting infections among many co-infected children suggest that routine multiplex testing may not always be necessary for clinical management. Conversely, pathogen combinations or specific detections associated with higher hospitalization risk may help identify children who benefit most from comprehensive diagnostic evaluation [30, 31]. Integrating these patterns into testing algorithms could support more targeted, efficient use of multiplex panels and strengthen diagnostic decision-making.

There were multiple limitations to this study. First, this study analyzed data from a single large tertiary pediatric hospital, so results may not be generalizable to other settings and pediatric populations. Second, patients who had stool samples tested may not represent all pediatric AGI cases, particularly cases in which patients had milder or asymptomatic AGI. Third, this study relied on electronic medical records, which limited the accessibility of relevant clinical details to visits within the NCH system and did not capture information on other factors (e.g., environmental exposures, clinical history) that could influence patient outcomes. Fourth, the CCI used to summarize comorbidities was developed for long-term mortality estimation in adults and may not accurately reflect acute comorbidity assessment in pediatric populations. Fifth, patient travel history could also be an important risk factor, but such information was not available. Finally, while the use of the BioFire® FilmArray® GI Panel enabled high-sensitivity detection of co-detections in single stool samples, co-detection of multiple pathogens does not necessarily indicate disease causation or severity. Despite these limitations, the results of this study provide valuable insights into the epidemiology of AGI in pediatric populations and offer information that can inform patient care and public health.

This study illuminates the complex dynamics of pathogen detection and co-testing for AGI in pediatric patients in tertiary care settings, highlighting the intricate interplay between sociodemographic factors and the likelihood of detecting multiple pathogens. This study highlights the intricate relationships between pathogen co-detection, patient demographics, and clinical outcomes, revealing the underlying mechanisms that drive these associations and informing the development of more targeted public health strategies. Although co-infected pediatric patients may be clinically vulnerable, the predominance of mild, self-limiting pathogens in these co-infections may explain why this group did not experience higher hospitalization rates. Further research is needed to clarify the clinical and public health implications of gastrointestinal co-infection in children.

## Supporting information

10.1017/S0950268826101083.sm001 Xiong et al. supplementary material Xiong et al. supplementary material

## Data Availability

The datasets generated and analyzed in the current study contain protected health information (PHI) and cannot be shared publicly to protect patient privacy. De-identified or limited datasets may be made available from the corresponding author on reasonable request, subject to approval by the appropriate institutional review boards and data use agreements.

## Supplementary material

The supplementary material for this article can be found at http://doi.org/10.1017/S0950268826101083.

## References

[1] Institute for Health Metrics and Evaluation (2020) *GBD Results.* https://vizhub.healthdata.org/gbd-results (accessed 16 April 2023).

[2] Torres-Miranda D, et al. (2020) Use of BioFire FilmArray gastrointestinal PCR panel associated with reductions in antibiotic use, time to optimal antibiotics, and length of stay. BMC Gastroenterology 20, 246. 32727381 10.1186/s12876-020-01394-w PMC7392718

[3] Beal SG, et al. (2018) A gastrointestinal PCR panel improves clinical management and lowers health care costs. Journal of Clinical Microbiology 56, e01457 17. 29093106 10.1128/JCM.01457-17 PMC5744222

[4] Janda JM and Abbott SA (2014) Culture-independent diagnostic testing: Have we opened Pandora’s box for good? Diagnostic Microbiology and Infectious Disease 80, 171–176. 25200256 10.1016/j.diagmicrobio.2014.08.001

[5] Ruzante JM, et al. (2021) Real-Time Gastrointestinal Infection Surveillance through a Cloud-Based Network of Clinical Laboratories. PLOS ONE Public Library of Science 16,e0250767. 10.1371/journal.pone.0250767 PMC8087049 33930062

[6] Buss SN, et al. (2013) Implications of culture-independent panel-based detection of Cyclospora cayetanensis. Journal of Clinical Microbiology American Society for Microbiology 51, 3909–3909. 10.1128/JCM.02238-13 PMC3889798 23985921

[7] Stockmann C, et al. (2015) How well does physician selection of microbiologic tests identify Clostridium difficile and other pathogens in paediatric diarrhoea? Insights using multiplex PCR-based detection. Clinical Microbiology and Infection: The Official Publication of the European Society of Clinical Microbiology and Infectious Diseases 21, 179.e9–179 e15. 10.1016/j.cmi.2014.07.011 PMC4330102 25599941

[8] Murphy CN, et al. (2017) Evaluation of the BioFire FilmArray® GastrointestinalPanel in a Midwestern academic hospital. European Journal of Clinical Microbiology & Infectious Diseases: Official Publication of the European Society of Clinical Microbiology 36, 747–754. 27957599 10.1007/s10096-016-2858-7

[9] Stockmann C, et al. (2016) Detection of 23 gastrointestinal pathogens among children who present with diarrhea. Journal of the Pediatric Infectious Diseases Society 6, 231–238. 10.1093/jpids/piw020 PMC5907859 27147712

[10] Valentini D, et al. (2013) Coinfection in acute gastroenteritis predicts a more severe clinical course in children. European Journal of Clinical Microbiology & Infectious Diseases: Official Publication of the European Society of Clinical Microbiology 32, 909–915. 23370970 10.1007/s10096-013-1825-9

[11] Zhang S-X, et al. (2016) Impact of co-infections with enteric pathogens on children suffering from acute diarrhea in Southwest China. Infectious Diseases of Poverty 5, 64. 27349521 10.1186/s40249-016-0157-2 PMC4922062

[12] Pijnacker R, et al. (2019) Clinical relevance of enteropathogen co-infections in preschool children—A population-based repeated cross-sectional study. Clinical Microbiology and Infection 25, 1039.e7–1039.e13. 10.1016/j.cmi.2018.11.029 30553029

[13] *(PDF) Coinfection in acute gastroenteritis predicts a more severe clinical course in children.* https://www.researchgate.net/publication/235391746_Coinfection_in_acute_gastroenteritis_predicts_a_more_severe_clinical_course_in_children?utm_source=chatgpt.com (accessed 21 November 2025). 10.1007/s10096-013-1825-9 23370970

[14] Yasmin N, et al. (2024) Detection of gastrointestinal pathogens in stool samples using a rapid multiplex PCR test at a large tertiary pediatric hospital. Diagnostic Microbiology and Infectious Disease 110, 116544. 39413661 10.1016/j.diagmicrobio.2024.116544

[15] BioFire Diagnostics. *FilmArray GI reagent instruction booklet - ITI0030.*

[16] *Rural-Urban Commuting Area Codes*. Economic Research Service. https://www.ers.usda.gov/data-products/rural-urban-commuting-area-codes (accessed 5 December 2025).

[17] Charlson ME, et al. (1987) A new method of classifying prognostic comorbidity in longitudinal studies: Development and validation. Journal of Chronic Diseases 40, 373–383. 3558716 10.1016/0021-9681(87)90171-8

[18] Cybulski RJ, et al. (2018) Clinical impact of a multiplex gastrointestinal polymerase chain reaction panel in patients with acute gastroenteritis. Clinical Infectious Diseases: An Official Publication of the Infectious Diseases Society of America 67, 1688–1696. 29697761 10.1093/cid/ciy357

[19] Mannstadt I, et al. (2024) Risk factors and clinical outcomes associated with multiple as opposed to single pathogens detected on the gastrointestinal disease polymerase chain reaction assay. Gut Pathogens 16, 45. 39215373 10.1186/s13099-024-00638-4 PMC11365154

[20] Spina A, et al. (2015) Spectrum of enteropathogens detected by the FilmArray GI panel in a multicentre study of community-acquired gastroenteritis. Clinical Microbiology and Infection 21, 719–728. 25908431 10.1016/j.cmi.2015.04.007

[21] De Grazia S, et al. (2020) Assessing the burden of viral co-infections in acute gastroenteritis in children: An eleven-year-long investigation. Journal of Clinical Virology 129, 104513. 32575023 10.1016/j.jcv.2020.104513

[22] Huerta-Franco M-R, Banderas JW and Allsworth JE (2018) Ethnic/racial differences in gastrointestinal symptoms and diagnosis associated with the risk of helicobacter pylori infection in the US. Clinical and Experimental Gastroenterology Dove Medical Press 11, 39–49. 10.2147/CEG.S144967 PMC5779296 29403299

[23] Olsen MA, et al. (2023) Trends in the incidence of Clostridioides difficile infection in adults and the elderly insured by Medicaid compared to commercial insurance or Medicare only. Infection Control & Hospital Epidemiology 44, 1076–1084. 36082779 10.1017/ice.2022.208 PMC9995604

[24] Mabbott NA (2018) The influence of parasite infections on host immunity to co-infection with other pathogens. Frontiers in Immunology Frontiers 9. 10.3389/fimmu.2018.02579. PMC6237250 30467504

[25] Ezenwa VO and Jolles AE (2011) From host immunity to pathogen invasion: The effects of helminth coinfection on the dynamics of microparasites. Integrative and Comparative Biology 51, 540–551. 21727178 10.1093/icb/icr058

[26] Cattadori IM, Boag B and Hudson PJ (2008) Parasite co-infection and interaction as drivers of host heterogeneity. International Journal for Parasitology 38, 371–380. 17936286 10.1016/j.ijpara.2007.08.004

[27] Lian S, et al. (2022) Bacterial and viral co-infection in the intestine: Competition scenario and their effect on host immunity. International Journal of Molecular Sciences Multidisciplinary Digital Publishing Institute 23, 2311. 10.3390/ijms23042311 PMC8877981 35216425

[28] Pavia AT, et al. (2023) Clinical impact of multiplex molecular diagnostic testing in children with acute gastroenteritis presenting to an emergency department: A multicenter prospective study. Clinical Infectious Diseases: An Official Publication of the Infectious Diseases Society of America 78, 573–581. 10.1093/cid/ciad710 PMC10954335 38097379

[29] Bizot E, et al. (2025) Use of gastrointestinal syndromic multiplex molecular assays and detection of Escherichia coli pathotypes in pediatric wards. Journal of Clinical Microbiology American Society for Microbiology 63, e01073–e01024. 10.1128/jcm.01073-24 PMC11980392 40008873

[30] Kang HM, Yoo IH and Jeong DC (2024) The role of rapid syndromic diagnostic testing of gastrointestinal pathogens as a clinical decision support tool in a pediatric emergency department. Annals of Clinical Microbiology and Antimicrobials 23, 3. 38183046 10.1186/s12941-023-00662-3 PMC10770992

[31] Chen C-H, et al. (2023) Rapid detection of gastrointestinal pathogens using a multiplex polymerase chain reaction gastrointestinal panel and its role in antimicrobial stewardship. Journal of Microbiology, Immunology and Infection 56, 1273–1283. 10.1016/j.jmii.2023.10.004 37926631

