# Supplementary material for: Patterns of gastrointestinal pathogen co-detection in pediatric stool samples identified by rapid multiplex PCR
Source: Epidemiol Infect. 2026 Feb 4;154:e24. doi: 10.1017/S0950268826101083 (PMC12951331; doi:10.1017/S0950268826101083)
Supplement: Xiong et al. supplementary material [file S0950268826101083sup001.zip › Supplementary Table 1.docx]

| Pathogen Combination | Count | Hospitalization rate^a^ |
| --- | --- | --- |
| *EAEC + EPEC* | 135 | 45.2 |
| *Norovirus + EPEC* | 92 | 58.7 |
| *Adenovirus + EPEC* | 67 | 53.7 |
| *Campylobacter + EPEC* | 65 | 64.6 |
| *C difficile + EPEC* | 59 | 40.7 |
| *Sapovirus + EPEC* | 53 | 47.2 |
| *Cryptosporidium + EPEC* | 43 | 51.2 |
| *Norovirus + EAEC* | 39 | 38.5 |
| *Rotavirus + EPEC* | 39 | 76.9 |
| *Norovirus + C difficile* | 30 | 73.3 |
| *Adenovirus + Sapovirus* | 30 | 40.0 |
| *Astrovirus + Norovirus* | 27 | 44.4 |
| *Sapovirus + EAEC* | 27 | 48.1 |
| *Norovirus + Sapovirus* | 26 | 50.0 |
| *Adenovirus + Norovirus* | 25 | 56.0 |
| *EPEC + Salmonella* | 25 | 48.0 |
| *EPEC + ETEC* | 25 | 48.0 |
| *Rotavirus + EAEC* | 25 | 60.0 |
| *Norovirus + Rotavirus* | 22 | 45.5 |
| *Astrovirus + Sapovirus* | 21 | 52.4 |
| *Rotavirus + C difficile* | 20 | 50.0 |
| *Norovirus + EAEC + EPEC* | 20 | 45.0 |
| *Rotavirus + Sapovirus* | 20 | 65.0 |
| *Adenovirus + EAEC* | 18 | 77.8 |
| *C difficile + EAEC* | 16 | 31.3 |
| *Shigella & EIEC + EPEC* | 15 | 60.0 |
| *EAEC + EPEC + ETEC* | 15 | 26.7 |
| *Astrovirus + Rotavirus* | 14 | 57.1 |
| *Adenovirus + EAEC + EPEC* | 13 | 46.2 |
| *Adenovirus + Astrovirus* | 13 | 61.5 |
| *Astrovirus + EPEC* | 12 | 58.3 |
| *Cryptosporidium + EAEC* | 12 | 33.3 |
| *Giardia + EPEC* | 11 | 45.5 |
| *C difficile + STEC/O157* | 10 | 40.0 |
| *Campylobacter + STEC/O157* | 10 | 70.0 |
| *Astrovirus + EAEC* | 10 | 40.0 |
| *Rotavirus + Salmonella* | 10 | 80.0 |
| *C difficile + Sapovirus* | 10 | 40.0 |
| *Cryptosporidium + Sapovirus* | 9 | 22.2 |
| *Campylobacter + C difficile* | 9 | 44.4 |
| *Adenovirus + Rotavirus* | 8 | 50.0 |
| *Shigella & EIEC + Sapovirus* | 8 | 62.5 |
| *Cryptosporidium + EAEC + EPEC* | 8 | 50.0 |
| *Astrovirus + C difficile* | 8 | 37.5 |
| *EAEC + Salmonella* | 8 | 0.0 |
| *Campylobacter + EAEC + EPEC* | 8 | 62.5 |
| *Shigella & EIEC + EAEC* | 7 | 42.9 |
| *Astrovirus + EAEC + EPEC* | 7 | 28.6 |
| *EPEC + Yersinia* | 7 | 42.9 |
| *EAEC + STEC/O157* | 7 | 28.6 |
| *C difficile + Salmonella* | 7 | 42.9 |
| *Shigella & EIEC + C difficile* | 6 | 50.0 |
| *Norovirus + Rotavirus + EPEC* | 6 | 66.7 |
| *Cryptosporidium + C difficile* | 6 | 33.3 |
| *Astrovirus + Norovirus + Sapovirus* | 6 | 50.0 |
| *Shigella & EIEC + Norovirus* | 6 | 83.3 |
| *Sapovirus + EAEC + EPEC* | 6 | 33.3 |
| *Campylobacter + EPEC + ETEC* | 6 | 16.7 |
| *EAEC + ETEC* | 6 | 33.3 |
| *Campylobacter + Adenovirus* | 5 | 20.0 |
| *Cryptosporidium + Rotavirus* | 5 | 40.0 |
| *Norovirus + Salmonella* | 5 | 20.0 |
| *Adenovirus + C difficile* | 5 | 80.0 |
| *Campylobacter + Sapovirus* | 5 | 60.0 |
| *C difficile + EAEC + EPEC* | 5 | 40.0 |
| *Cryptosporidium + Salmonella* | 5 | 80.0 |
| *Rotavirus + EAEC + EPEC* | 5 | 80.0 |
| *Campylobacter + Norovirus* | 4 | 50.0 |
| *Cryptosporidium + Giardia + EPEC* | 4 | 25.0 |
| *Astrovirus + Sapovirus + EAEC* | 4 | 50.0 |
| *Adenovirus + STEC/O157* | 4 | 50.0 |
| *Shigella & EIEC + EAEC + EPEC* | 4 | 75.0 |
| *Cryptosporidium + Sapovirus + EPEC* | 4 | 25.0 |
| *Adenovirus + Rotavirus + EAEC* | 4 | 75.0 |
| *EPEC + Plesiomonas* | 4 | 75.0 |
| *EAEC + EPEC + Salmonella* | 4 | 0.0 |
| *Giardia + Norovirus* | 4 | 50.0 |
| *Cryptosporidium + STEC/O157* | 4 | 75.0 |
| *Campylobacter + Rotavirus* | 4 | 50.0 |
| *Giardia + Astrovirus* | 4 | 75.0 |
| *Salmonella + ETEC* | 4 | 100.0 |
| *Adenovirus + Norovirus + EAEC + EPEC* | 3 | 0.0 |
| *STEC/O157 + EAEC* | 3 | 33.3 |
| *Campylobacter + Astrovirus* | 3 | 33.3 |
| *Shigella & EIEC + EPEC + Plesiomonas* | 3 | 66.7 |
| *Campylobacter + ETEC* | 3 | 66.7 |
| *Adenovirus + Salmonella* | 3 | 100.0 |
| *Adenovirus + EPEC + Salmonella* | 3 | 66.7 |
| *Adenovirus + Sapovirus + EPEC* | 3 | 0.0 |
| *Adenovirus + Norovirus + EAEC* | 3 | 0.0 |
| *Giardia + EAEC* | 3 | 66.7 |
| *Norovirus + ETEC* | 3 | 66.7 |
| *Campylobacter + EAEC* | 3 | 33.3 |
| *Adenovirus + Astrovirus + EPEC* | 3 | 33.3 |
| *Rotavirus + Sapovirus + EPEC* | 3 | 0.0 |
| *Giardia + C difficile* | 3 | 33.3 |
| *Rotavirus + STEC/O157* | 3 | 66.7 |
| *Campylobacter + C difficile + EPEC* | 3 | 33.3 |
| *Sapovirus + ETEC* | 3 | 0.0 |
| *Campylobacter + Rotavirus + EPEC* | 3 | 66.7 |
| *Giardia + EAEC + EPEC* | 3 | 33.3 |
| *Shigella & EIEC + Cryptosporidium + EPEC* | 3 | 33.3 |
| *Campylobacter + Giardia* | 3 | 33.3 |
| *Rotavirus + Yersinia* | 3 | 100.0 |
| *Cryptosporidium + EPEC + ETEC* | 3 | 33.3 |

^a^ Percentage of patients with selected pathogen combination that

were hospitalized
