# Supplementary material for: Patterns of gastrointestinal pathogen co-detection in pediatric stool samples identified by rapid multiplex PCR
Source: Epidemiol Infect. 2026 Feb 4;154:e24. doi: 10.1017/S0950268826101083 (PMC12951331; doi:10.1017/S0950268826101083)
Supplement: Xiong et al. supplementary material [file S0950268826101083sup001.zip › Supplementary Table 2.docx]

**Additional File 1: Logistic regressions of risk factors associated with co-detection, hospitalization, and death in patients, 2016-2020**

| **Co-detections** | | | | | |
| --- | --- | --- | --- | --- | --- |
| **Factor** | | **COR^a^ (95% CI^b^)** | **p-value** | **AOR^c^ (95% CI)** | **p-value** |
| Age group | 1-4 vs 13-18 | 2.2 (1.7, 2.9) | <0.0001 | 2.1 (1.6, 2.7) | <0.0001 |
|  | 1-4 vs 5-12 | 1.6 (1.3, 1.9) |  | 1.5 (1.2, 1.8) |  |
|  | 1-4 vs <1 | 1.1 (0.9, 1.4) |  | 1.2 (0.9, 1.4) |  |
|  | 1-4 vs >18 | 2.1 (1.3, 3.5) |  | 1.9 (1.1, 3.2) |  |
|  | 13-18 vs 5-12 | 0.7 (0.5, 0.9) |  | 0.7 (0.5, 1.0) |  |
|  | 13-18 vs <1 | 0.5 (0.4, 0.7) |  | 0.6 (0.4, 0.8) |  |
|  | 13-18 vs >18 | 0.9 (0.5, 1.6) |  | 0.9 (0.5, 1.6) |  |
|  | 5-12 vs <1 | 0.7 (0.6, 0.9) |  | 0.8 (0.6, 1.0) |  |
|  | 5-12 vs >18 | 1.3 (0.8, 2.3) |  | 1.3 (0.8, 2.2) |  |
|  | <1 vs >18 | 1.9 (1.1, 3.2) |  | 1.6 (1.0, 2.8) |  |
| Race | Asian vs Black Non-Hispanic | 1.2 (0.8, 1.7) | <0.0001 | 1.2 (0.8, 1.8) | <0.0001 |
|  | Asian vs Hispanic or Latino | 1.0 (0.6, 1.5) |  | 1.0 (0.6, 1.7) |  |
|  | Asian vs Other | 1.3 (0.8, 2.0) |  | 1.3 (0.8, 2.0) |  |
|  | Asian vs White Non-Hispanic | 1.7 (1.2, 2.5) |  | 1.5 (1.1, 2.3) |  |
|  | Black Non-Hispanic vs Hispanic or Latino | 0.8 (0.6, 1.2) |  | 0.8 (0.6, 1.2) |  |
|  | Black Non-Hispanic vs Other | 1.1 (0.8, 1.5) |  | 1.1 (0.8, 1.5) |  |
|  | Black Non-Hispanic vs White Non-Hispanic | 1.5 (1.2, 1.8) |  | 1.3 (1.0, 1.6) |  |
|  | Hispanic or Latino vs Other | 1.3 (0.9, 2.0) |  | 1.3 (0.9, 1.9) |  |
|  | Hispanic or Latino vs White Non-Hispanic | 1.8 (1.3, 2.5) |  | 1.5 (1.1, 2.1) |  |
|  | Other vs White Non-Hispanic | 1.3 (1.0, 1.8) |  | 1.2 (0.9, 1.6) |  |
| Insurance group | Combination vs Private | 0.8 (0.6, 1.1) | <0.0001 | 0.8 (0.6, 1.1) | <0.0001 |
|  | Combination vs Public | 0.5 (0.4, 0.7) |  | 0.6 (0.4, 0.9) |  |
|  | Combination vs Self-pay | 0.4 (0.2, 0.7) |  | 0.5 (0.3, 0.8) |  |
|  | Private vs Public | 0.7 (0.6, 0.8) |  | 0.8 (0.7, 1.0) |  |
|  | Private vs Self-pay | 0.5 (0.3, 0.9) |  | 0.6 (0.4, 1.0) |  |
|  | Public vs Self-pay | 0.8 (0.5, 1.3) |  | 0.8 (0.5, 1.3) |  |
| Gender | Female vs Male | 1.0 (0.9, 1.1) | 0.7007 | - | - |
| Urbanicity | Metropolitan Area vs Micropolitan Area | 1.1 (0.9, 1.4) | 0.3823 | - | - |
|  | Metropolitan Area vs Missing | 0.6 (0.2, 2.8) |  | - |  |
|  | Metropolitan Area vs Rural Area | 1.3 (0.6, 2.7) |  | - |  |
|  | Metropolitan Area vs Small Town | 0.9 (0.6, 1.3) |  | - |  |
|  | Micropolitan Area vs Missing | 0.6 (0.1, 2.5) |  | - |  |
|  | Micropolitan Area vs Rural Area | 1.1 (0.5, 2.5) |  | - |  |
|  | Micropolitan Area vs Small Town | 0.8 (0.5, 1.2) |  | - |  |
|  | Missing vs Rural Area | 1.9 (0.4, 10.1) |  | - |  |
|  | Missing vs Small Town | 1.3 (0.3, 6.0) |  | - |  |
|  | Rural Area vs Small Town | 0.7 (0.3, 1.7) |  | - |  |
| **Hospitalization** | | | | | |
| **Factor** | | **COR^a^ (95% CI^b^)** | **p-value** | **AOR^c^ (95% CI)** | **p-value** |
| Age group | 1-4 vs 13-18 | 1.3 (1.1, 1.5) | <0.0001 | 1.2 (1.0, 1.4) | <0.0001 |
|  | 1-4 vs 5-12 | 1.0 (0.9, 1.2) |  | 1.0 (0.9, 1.2) |  |
|  | 1-4 vs <1 | 0.5 (0.4, 0.6) |  | 0.5 (0.4, 0.6) |  |
|  | 1-4 vs >18 | 1.2 (1.0, 1.5) |  | 1.1 (0.9, 1.4) |  |
|  | 13-18 vs 5-12 | 0.8 (0.7, 1.0) |  | 0.8 (0.7, 1.0) |  |
|  | 13-18 vs <1 | 0.4 (0.3, 0.5) |  | 0.4 (0.3, 0.5) |  |
|  | 13-18 vs >18 | 1.0 (0.8, 1.2) |  | 0.9 (0.7, 1.2) |  |
|  | 5-12 vs <1 | 0.5 (0.4, 0.6) |  | 0.5 (0.4, 0.6) |  |
|  | 5-12 vs >18 | 1.2 (0.9, 1.5) |  | 1.1 (0.9, 1.4) |  |
|  | <1 vs >18 | 2.4 (1.9, 3.1) |  | 2.2 (1.7, 2.9) |  |
| Race | Asian vs Black Non-Hispanic | 0.6 (0.5, 0.9) | <0.0001 | 0.7 (0.5, 0.9) | <0.0001 |
|  | Asian vs Hispanic or Latino | 0.6 (0.5, 0.9) |  | 0.9 (0.7, 1.3) |  |
|  | Asian vs Other | 0.4 (0.3, 0.5) |  | 0.5 (0.3, 0.6) |  |
|  | Asian vs White Non-Hispanic | 0.6 (0.5, 0.8) |  | 0.7 (0.6, 1.0) |  |
|  | Black Non-Hispanic vs Hispanic or Latino | 1.4 (1.2, 1.7) |  | 1.4 (1.1, 1.8) |  |
|  | Black Non-Hispanic vs Other | 0.6 (0.5, 0.7) |  | 0.7 (0.5, 0.8) |  |
|  | Black Non-Hispanic vs White Non-Hispanic | 1.0 (0.9, 1.1) |  | 1.1 (0.9, 1.3) |  |
|  | Hispanic or Latino vs Other | 0.4 (0.4, 0.5) |  | 0.5 (0.4, 0.7) |  |
|  | Hispanic or Latino vs White Non-Hispanic | 0.7 (0.6, 0.8) |  | 0.8 (0.6, 1.0) |  |
|  | Other vs White Non-Hispanic | 1.6 (1.5, 1.9) |  | 1.6 (1.3, 1.9) |  |
| Insurance group | Combination vs Private | 1.9 (1.7, 2.2) | <0.0001 | 1.9 (1.6, 2.3) | <0.0001 |
|  | Combination vs Public | 1.3 (1.1, 1.5) |  | 1.4 (1.1, 1.6) |  |
|  | Combination vs Self-pay | 1.5 (1.1, 2.0) |  | 1.5 (1.0, 2.2) |  |
|  | Private vs Public | 0.7 (0.6, 0.7) |  | 0.7 (0.6, 0.8) |  |
|  | Private vs Self-pay | 0.8 (0.6, 1.0) |  | 0.8 (0.6, 1.1) |  |
|  | Public vs Self-pay | 1.2 (0.9, 1.5) |  | 1.1 (0.8, 1.5) |  |
| Gender | Female vs Male | 1.0 (0.9, 1.1) | 0.8509 | - | - |
| Urbanicity | Metropolitan Area vs Micropolitan Area | 0.4 (0.4, 0.4) | <0.0001 | 0.4 (0.3, 0.5) | <0.0001 |
|  | Metropolitan Area vs Missing | 0.1 (0.1, 0.3) |  | 0.2 (0.1, 1.0) |  |
|  | Metropolitan Area vs Rural Area | 0.4 (0.3, 0.6) |  | 0.4 (0.3, 0.7) |  |
|  | Metropolitan Area vs Small Town | 0.3 (0.2, 0.4) |  | 0.3 (0.2, 0.4) |  |
|  | Micropolitan Area vs Missing | 0.3 (0.1, 0.8) |  | 0.6 (0.1, 2.6) |  |
|  | Micropolitan Area vs Rural Area | 1.1 (0.8, 1.5) |  | 1.0 (0.6, 1.7) |  |
|  | Micropolitan Area vs Small Town | 0.8 (0.6, 1.0) |  | 0.8 (0.6, 1.1) |  |
|  | Missing vs Rural Area | 3.4 (1.2, 9.2) |  | 1.7 (0.4, 7.9) |  |
|  | Missing vs Small Town | 2.4 (0.9, 6.4) |  | 1.3 (0.3, 5.6) |  |
|  | Rural Area vs Small Town | 0.7 (0.5, 1.1) |  | 0.7 (0.4, 1.4) |  |
| Co-detection | No detection vs Single detection | 1.0 (0.9, 1.1) | 0.2589 | - | -  -  - |
|  | No detection vs Co-detection | 1.1 (1.0, 1.2) |  | - |  |
|  | Single detection vs Co-detection | 1.1 (1.0, 1.2) |  | - |  |
| **Death** | | | | | |
| **Factor** | | **COR^a^ (95% CI^b^)** | **p-value** | **AOR^c^ (95% CI)** | **p-value** |
| Age group | 1-4 vs 13-18 | 0.5 (0.1, 2.1) | <0.0001 | LCC^d^ | - |
|  | 1-4 vs 5-12 | 0.2 (0.1, 0.7) |  | LCC | - |
|  | 1-4 vs <1 | 0.1 (0.0, 0.3) |  | LCC | - |
|  | 1-4 vs >18 | 0.1 (0.0, 0.2) |  | LCC | - |
|  | 13-18 vs 5-12 | 0.4 (0.1, 1.2) |  | LCC | - |
|  | 13-18 vs <1 | 0.2 (0.1, 0.5) |  | LCC | - |
|  | 13-18 vs >18 | 0.1 (0.0, 0.3) |  | LCC | - |
|  | 5-12 vs <1 | 0.4 (0.2, 0.9) |  | 0.3 (0.1, 0.9) | 0.0078 |
|  | 5-12 vs >18 | 0.3 (0.1, 0.6) |  | 0.3 (0.1, 0.8) |  |
|  | <1 vs >18 | 0.6 (0.3, 1.4) |  | 0.8 (0.3, 2.1) |  |
| Race | Black Non-Hispanic vs Other | 0.9 (0.4, 2.1) | 0.0850 | - | - |
|  | Black Non-Hispanic vs White Non-Hispanic | 1.8 (0.9, 3.6) |  | - |  |
|  | Other vs White Non-Hispanic | 2.1 (0.9, 4.6) |  | - |  |
| Insurance group | Combination vs Private | 14.6 (5.4, 39.7) | <0.0001 | LCC | - |
|  | Combination vs Public | 3.8 (2.0, 7.0) |  | LCC | - |
|  | Private vs Public | 0.3 (0.1, 0.7) |  | LCC | - |
| Gender | Female vs Male | 1.0 (0.5, 1.7) | 0.9107 | - | - |
| Urbanicity | Metropolitan area vs Micropolitan area | 0.7 (0.4, 1.3) | 0.2817 | - | - |
| Co-detection | No detection vs Single detection | 2.4 (1.2, 4.6) | 0.0126 | 2.4 (1.1, 5.2) | 0.0316 |

^a^ Crude odds ratio

^b^ 95% Wald Confidence Interval with Bonferroni adjustment

^c^  Adjusted odds ratio

^d^ Low cell count
