# Supplementary figures and images for: Patterns of gastrointestinal pathogen co-detection in pediatric stool samples identified by rapid multiplex PCR
Source: Epidemiol Infect. 2026 Feb 4;154:e24. doi: 10.1017/S0950268826101083 (PMC12951331; doi:10.1017/S0950268826101083)

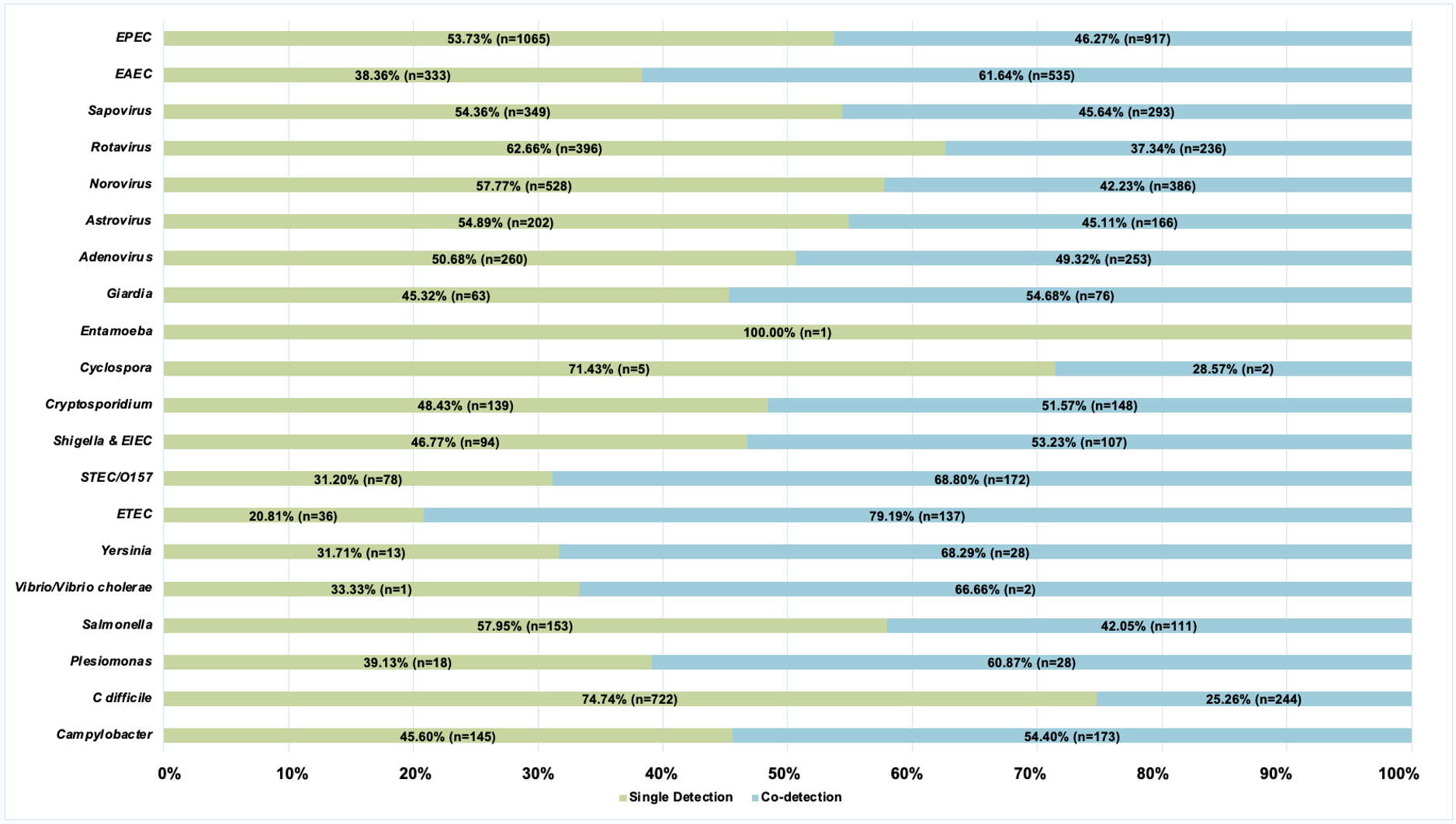

Supplement: Xiong et al. supplementary material [file S0950268826101083sup001.zip › Supplementary Figure 1.tiff]
